# Advanced Bearing-Fault Diagnosis and Classification Using Mel-Scalograms and FOX-Optimized ANN

**DOI:** 10.3390/s24227303

**Published:** 2024-11-15

**Authors:** Muhammad Farooq Siddique, Wasim Zaman, Saif Ullah, Muhammad Umar, Faisal Saleem, Dongkoo Shon, Tae Hyun Yoon, Dae-Seung Yoo, Jong-Myon Kim

**Affiliations:** 1 Department of Electrical, Electronics and Computer Engineering, University of Ulsan, Ulsan 44610, Republic of Korea; mfarooq229@mail.ulsan.ac.kr (M.F.S.); saifuou@mail.ulsan.ac.kr (S.U.); muhammadumar@mail.ulsan.ac.kr (M.U.);; 2 Electronics and Telecommunications Research Institute (ETRI), Daejeon 34129, Republic of Koreathyoon0820@etri.re.kr (T.H.Y.); ooseyds@etri.re.kr (D.-S.Y.)

**Keywords:** vibrational signals (VS), fault diagnosis, MEL-spectrum, artificial neural network, fox optimizer

## Abstract

Accurate and reliable bearing-fault diagnosis is important for ensuring the efficiency and safety of industrial machinery. This paper presents a novel method for bearing-fault diagnosis using Mel-transformed scalograms obtained from vibrational signals (VS). The signals are windowed and pass through a Mel filter bank, converting them into a Mel spectrum. These scalograms are subsequently fed into an autoencoder comprising convolutional and pooling layers to extract robust features. The classification is performed using an artificial neural network (ANN) optimized with the FOX optimizer, which replaces traditional backpropagation. The FOX optimizer enhances synaptic weight adjustments, leading to superior classification accuracy, minimal loss, improved generalization, and increased interpretability. The proposed model was validated on a laboratory dataset obtained from a bearing testbed with multiple fault conditions. Experimental results demonstrate that the model achieves perfect precision, recall, F1-scores, and an AUC of 1.00 across all fault categories, significantly outperforming comparison models. The t-SNE plots illustrate clear separability between different fault classes, confirming the model’s robustness and reliability. This approach offers an efficient and highly accurate solution for real-time predictive maintenance in industrial applications.

## 1. Introduction

With the rapid advancement of modern industrial systems, rotating machinery has become essential to intelligent equipment, drawing considerable attention from both academia and industry [1]. Critical transmission components in rotating machinery, such as bearings and gears, are exposed to wear, corrosion, deformation, cracking, and other types of failure in demanding operating conditions, including heavy loads and high speeds [2]. Faults in these components directly impact the operational reliability of rotating machinery and can lead to severe accidents, causing substantial economic losses and even posing risks to human safety [3]. Consequently, fault diagnosis and predictive maintenance of rotating machinery hold significant research importance [4,5].

Researchers have explored various aspects of condition monitoring, including fault detection and its classification [6], severity diagnosis, and prognosis [7,8]. These methods require expertise, are time-consuming, expensive, and often lack high accuracy [9]. Fault diagnosis methods include reactive, preventive, and predictive maintenance, with reactive being the costliest. Approaches rely on quantitative models, qualitative models, or data-driven methods. Due to advances in technology, data-driven methods, which involve data acquisition, preprocessing, feature extraction–selection, and classification, have become preferred [10]. The diagnosis of induction motor faults is primarily conducted using various signals acquired from motors, including VS, acoustic emission (AE) signals, motor currents, temperature, and thermal images. In vibration-signal-based methods, vibration sensors collect the signal, which is then analyzed for fault investigation. Sensors are placed around the bearings under investigation, requiring constant access for data recording. Although vibration data contain extensive information on mechanical faults, accurately extracting fault characteristic signals is essential due to the noisy environment surrounding the components [11]. The vibration signal is an indicator of the operational status of a rotating mechanical structure, making state recognition a matter of classifying these signals. The process includes data preprocessing, feature extraction, pattern recognition, and model application, with feature extraction and algorithm identification being the main steps [12]. Features can be derived from time, frequency, time–frequency, or other transformed domains. In the time domain, common indicators include peak value and variance, while the frequency domain uses techniques like Fourier transform [13]. Time–frequency analysis provides simultaneous insights, employing methods such as short-time Fourier transform (STFT) and wavelet transform [14]. Since both domains reflect faulty information, real-time changes are important. The STFT converts signals into a time–frequency spectrum, with low frequencies being particularly informative. Compared to traditional diagrams, the Mel spectrum offers higher resolution in the low-frequency band, improving classification model performance [15]. The Mel spectrum, originally used in audio processing, is highly effective for mechanical fault diagnosis due to its focus on lower frequencies, which often reveal critical fault-related information [16]. This emphasis aligns with human auditory perception, where lower frequencies are more prominent, making it advantageous for bearing-fault diagnosis, as many fault-induced vibrations occur within this range. The Mel transformation applies a non-linear scaling via a Mel filter bank, enhancing lower frequencies while compressing higher ones, resulting in a time–frequency representation that captures subtle diagnostic features efficiently. To calculate the Mel spectrum, the signal is divided into frames, windowed, transformed with a Fourier transform, filtered through a Mel filter bank, and subjected to a logarithmic transformation [17]. This process creates a Mel scalogram that is compact and low-dimensional, retaining essential fault information while minimizing noise and computational demands, making it ideal for real-time monitoring. Unlike wavelet transforms, which provide adaptable, multi-resolution analysis but require more computational resources, the Mel spectrum offers a fixed, computationally efficient low-frequency emphasis that is particularly useful for applications focused on low-frequency fault detection [18]. This study, therefore, employs the Mel spectrum for feature extraction in bearing diagnostics.

In the era of intelligent fault diagnosis, data-driven methods have gained significant attention due to their high level of automation. Han et al. [19] proposed a method with strong generalization capabilities, ensuring it works under unseen conditions by regularizing both intrinsic and extrinsic generalization objectives. Recently, the advent of deep learning has further advanced fault diagnosis as an extension of data-driven approaches. Convolutional neural networks (CNNs) have been widely used for their ability to accurately and automatically extract features [20,21]. For instance, Cao et al. [22] developed an unsupervised domain-share CNN to extract domain-invariant features for fault diagnosis under varying rotational speeds. Zhou et al. [23] introduced a fault diagnosis framework based on Bayesian deep learning to make fault data more comprehensible and address domain shift issues. Shao et al. [24] proposed a method combining parameter transfer and infrared thermal images for fault diagnosis at variable rotating speeds. Janssens et al. [25] applied CNNs to one-dimensional time-domain signals, automatically extracting features of various bearing-fault types and lubrication degradation patterns. Zhang et al. [26] introduced a novel CNN-based framework incorporating dropout and ensemble learning techniques to enhance noise resistance and domain adaptation. Other approaches have leveraged CNNs’ image-processing capabilities by combining them with 2D signal representations. Ding and He [27] proposed an energy-fluctuating multiscale feature-mining approach using wavelet packet energy images and CNNs for spindle bearings. Wen et al. [28] introduced a CNN method based on Lenet-5, tested on various datasets from a bearing self-priming centrifugal pump and an axial piston hydraulic pump. Using 1D input data, Abdeljaber et al. [29] proposed a CNN-based method that integrates feature extraction and classification into a single block for real-time diagnosis. As computational resources have advanced, deep learning has gained prominence in prognostics due to its effectiveness in modelling complex systems. ANNs are the most used deep-learning methods for prognostics, thanks to their excellent performance in handling complex, non-linear, multi-dimensional systems [30]. Their robustness against noise and ability to process non-linear information make them particularly effective. Various ANN architectures, including Feed-forward, Single and Multilayer Perceptron [31], Recurrent Neural Networks (RNNs) [32], Long Short-Term Memory (LSTM) [33], Modular Neural Networks (MNNs) [34], and CNNs [35], are utilized for condition-based maintenance (CBM) and prognostics in industrial and renewable energy systems. For diagnosing bearing faults, a large memory storage retrieval (LAMSTAR) neural network based on an optimized deep-learning structure has been proposed [36]. Additionally, a Bayesian deep-learning model is used to characterize the latent structure between remaining useful life (RUL) and degradation features, describing prognostic uncertainties [37].

The traditional techniques often struggle with achieving high accuracy and generalizability due to the complexity and variability of VS. Many models rely on backpropagation-based methods that can be prone to overfitting and may lack robustness, particularly when working with limited datasets or when extracting meaningful features from complex signal data [38]. Additionally, the non-linear nature of VS. can result in the loss of critical fault characteristics during feature extraction, further limiting the effectiveness of traditional models. Furthermore, signal-processing methods may not capture essential characteristics needed for accurate fault classification, leading to suboptimal model performance. To address these challenges, this study introduces a novel approach leveraging Mel-transformed scalograms, which preserve both time- and frequency-domain information, to enhance the feature extraction and classification of bearing faults. By integrating a specialized autoencoder and a FOX optimizer for classification, the proposed model aims to overcome the limitations of conventional methods, providing improved robustness and generalizability.

The novelty and main contributions of the current study are as follows:

(1) A new type of Mel-transformed scalogram derived from vibration signals. This process involves windowing the signals and applying a Mel filter bank, transforming them into Mel-spectra that highlight essential fault-related features, often missed by traditional signal-processing methods.
(2) The generated Mel scalograms are then input into an autoencoder’s convolutional and pooling layers, enabling efficient extraction of meaningful features specific to fault detection directly from the Mel spectrum.
(3) For classification, an ANN is employed, utilizing the FOX optimizer in place of traditional backpropagation. This approach improves accuracy, reduces loss, enhances generalization, and offers better interpretability, addressing limitations present in previous optimization methods.
(4) The model’s effectiveness is rigorously validated on a bearing-testbed dataset featuring diverse fault conditions, demonstrating its robustness and generalizability across multiple fault types, highlighting the model’s potential for real-world fault diagnosis applications.
(5) Experimental results showcase the proposed model’s robustness and generalizability, making it a promising solution for complex fault detection tasks in bearing systems.

The structure of this paper is organized as follows: Section 2 describes the proposed methodology of the study, followed by findings presented in Section 3. Finally, Section 4 offers the study conclusion.

## 2. Proposed Method for Fault Diagnosis in Bearings

**Figure 1:**
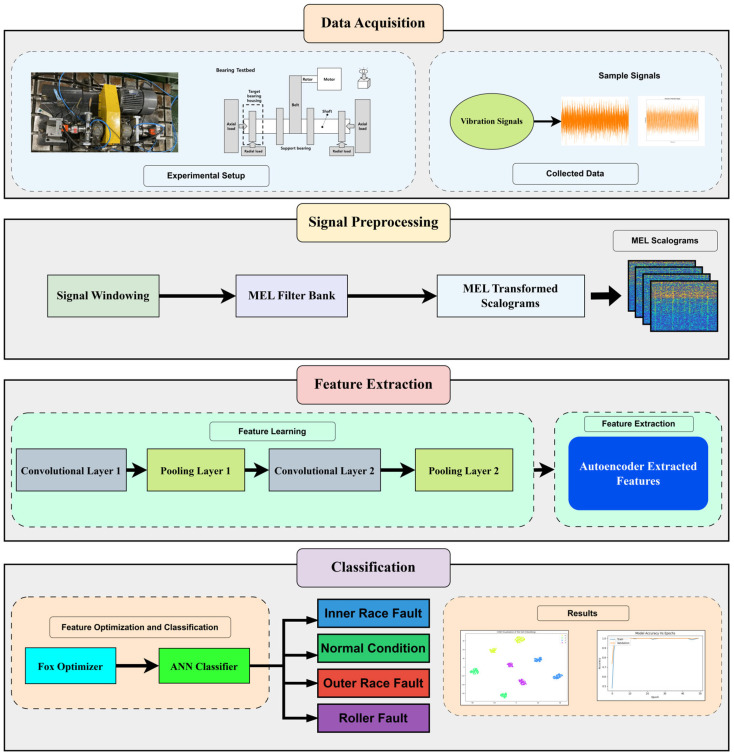
The complete workflow of the proposed model for bearing-fault detection.

The proposed method leverages Mel-transformed scalograms to optimize the feature extraction process in bearing-fault diagnosis. By focusing on low-frequency characteristics via the Mel transformation, the model achieves an efficient and accurate fault detection system across various fault types. These Mel scalograms are further processed through an autoencoder, which refines high-level features, followed by classification through an ANN optimized with the FOX algorithm. This novel combination enhances the diagnostic accuracy, robustness, and generalization of the model, making it a promising solution for complex fault detection tasks in industrial settings. Figure 1 depicts the complete workflow of the proposed model. The proposed method can be explained as follows:

(1) Data Acquisition and Signal Preprocessing: VSs from a bearing testbed are collected and preprocessed by windowing the signals and applying a wavelet transform. These windowed signals are then passed through a Mel filter bank to generate Mel-transformed scalograms, representing the time–frequency characteristics of the signals.
(2) Feature Extraction using Autoencoder: The generated Mel scalograms are fed into an autoencoder with two convolutional and two pooling layers for feature extraction. The autoencoder effectively captures significant high-level features from the scalograms while reducing dimensionality, ensuring that essential fault-relevant patterns are retained.
(3) Classification with FOX Optimizer and ANN: The extracted features are passed to an ANN, where the classification is optimized using the FOX optimizer. This optimizer replaces traditional backpropagation, improving accuracy, minimizing loss, and enhancing generalization. The model categorizes faults into four classes: Inner Race Fault (IRF), Outer Race Fault (ORF), Roller Fault (RF), and Normal Condition (NC).
(4) Model Evaluation and Validation: The proposed model is validated using experimental data from the bearing testbed. The results demonstrate the robustness and generalization ability of the model, achieving accurate fault detection across various fault conditions. Visualizations, including confusion matrices and accuracy curves, showcase the model’s effectiveness in fault classification.

### 2.1. Mel Transformation

**Figure 2:**
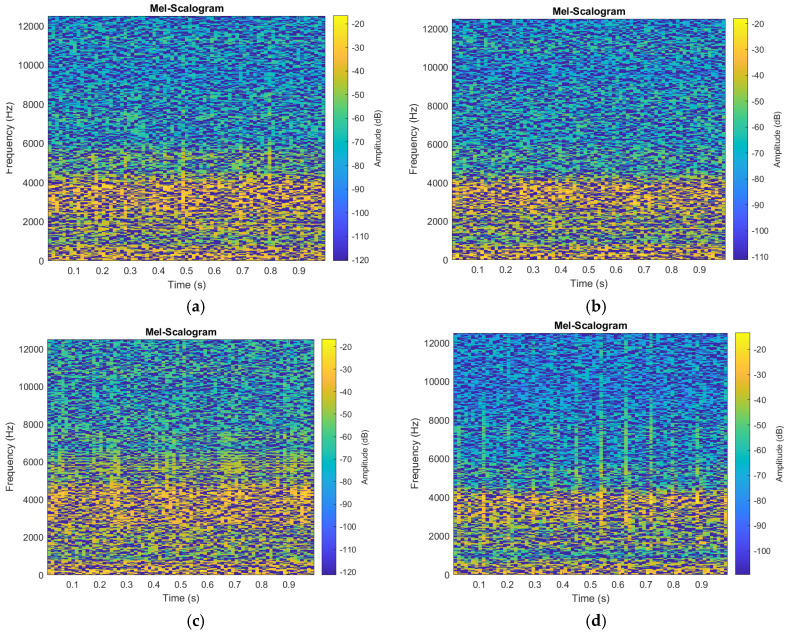
Mel-transformed scalograms of bearing faults: (**a**) IRF (**b**) NC (**c**) ORF (**d**) RF.

There are numerous methods for signal feature extraction, including those based on the time domain, frequency domain, and time–frequency domain. Figure 2 demonstrates that the vibration signal contains distinctive information in both the time and frequency domains. Consequently, a time–frequency domain feature extraction method is used. The Mel spectrum is a powerful tool for classifying signals such as speech, music, and VS. To calculate the Mel spectrum, several steps are required: frame division, windowing, Fourier transform, Mel filter bank, and logarithmic transformation. First, the signal is divided into several frames of a specified length. Each frame is then processed with a window function. The windowed frame signal is converted to the frequency domain using the Fourier transform. This frequency domain signal is then filtered through the Mel filter bank, converting it into the Mel frequency domain. Finally, the logarithm of the output from each Mel filter is computed to obtain the Mel spectrum. The Mel spectrum is like the scalogram produced by the short-time Fourier transform, with the key difference being the conversion of the ordinary frequency scale into the Mel-frequency scale. The following steps illustrate the whole transformation process.

(1) Vibration data are loaded from the dataset and extract relevant parameters, such as the signal data (s) and sampling frequency fs.
(2) The p-spectrum function computes the time–frequency representation (spectrogram) of the signal (s). This can be represented mathematically as follows:

(1) $$S(t,f)={|STFT(s(t))|}^{2}$$

where

  - *S* (*t, f*) is the spectrogram, representing energy distribution over time t and frequency f, and *s*(*t*) is the time-domain signal to be transformed.
  - Frequency limits in p-spectrum define the range [0, fs/2], limiting the analysis to frequencies within the Nyquist limit.

(3) The Mel transformation is implied in the choice of plotting the frequency spectrum with emphasis on lower-frequency bands, even though the exact formula is not directly used in your code. The formula for mapping frequency to the Mel scale is as follows:

(2) $$m=2595\cdot {log}_{10}(1+700f)$$

(4) While the Mel filter bank is not directly implemented, p-spectrum helps achieve a similar effect by emphasizing certain frequency ranges. The frequency resolution parameter essentially dictates the resolution of the time–frequency representation, allowing the low-frequency bands (where bearing fault characteristics are more likely to be found) to be more pronounced.
(5) A visual representation of the Mel-transformed scalogram for each class is generated with all labels as attached in Figure 2.

External interference primarily affects the low-frequency band of VS. The Mel spectrum enhances the resolution of these signals in the low-frequency band by applying the Mel filter bank and logarithmic transformation. This enhanced resolution aids in more effective feature extraction and improves the effectiveness of neural network models in fault diagnosis.

### 2.2. Convolutional Autoencoders (CAEs)

CAEs have become increasingly prominent in recent years due to their ability to learn complex, hierarchical representations of data, particularly in image-processing tasks. First introduced by Theis et al. [39] and Balle et al. [40], CAEs are a specialized type of neural network specifically designed to handle spatially structured inputs, such as images. Unlike traditional autoencoders that rely on fully connected layers, CAEs use convolutional layers to exploit the spatial relationships within the data, making them highly effective for various image-related applications like noise reduction, feature extraction, and compression. The primary objective of a CAE is to reconstruct the input as accurately as possible but under certain constraints, such as a limited number of neurons in the hidden layers. This limitation forces the model to focus on learning essential features and filtering out redundant information, thereby leading to more efficient representations. The CAE architecture comprises an encoder and a decoder; the encoder compresses the input, $x\epsilon R^{n}$, into a smaller latent space, capturing critical information in a condensed form. Here, the input consists of n feature maps of size l × l pixels, derived from the initial convolutional layer, where each map highlights different aspects of the image. The decoder then reconstructs the original input from this latent representation, aiming to replicate it as closely as possible. During this process, the output layer generates m feature maps through convolutional kernels with dimensions d × d, where d is less than or equal to 1. By applying these kernels, the CAE learns to identify spatial hierarchies and intricate patterns within the input data, which makes it particularly useful for tasks such as classification, anomaly detection, and as a pre-processing step for more complex models in computer vision. The capability of CAEs to perform dimensionality reduction and learn features in an unsupervised manner has led to their widespread adoption in deep learning and image processing, where their effectiveness in extracting meaningful representations continues to make them a valuable tool.

The process starts by encoding the input image, which is divided into d × d pixel patches, denoted as $x_{i}$, where (*i* = 1, 2, 3, …, *p*). For each of these patches, features from the input image are extracted, and convolution operations are performed using the weight $w_{j}$ of $j^{th}$ the convolutional kernel. This operation produces neuron values $O_{ij}$ for *j* = 1, 2, 3, …. *m* in the output layer.

(3) $$O_{ij}=f\left(x_{i}\right)=\sigma (w_{j}+b)$$

The non-linear activation function is denoted by the symbol σ. In this study, the rectified linear unit (ReLU) activation function is employed, which is defined as follows:

(4) $$ReLU\left(x\right)=\int \begin{array}{c} x\;if\;x\geq 0\\ 0\;if\;x<0 \end{array}$$

This function introduces nonlinearity into the model, allowing it to learn complex patterns by activating neurons only when the input is positive.

After the convolutional decoder produces the output $O_{ij}$ during the encoding process, the reconstruction of the original input $x_{i}$ is achieved using $O_{ij}$. This process results in the reconstructed output $x_{i}$, which aims to closely approximate the original input patch.

(5) $$x_{i}=f^{\prime }\left(O_{ij}\right)=\emptyset (w_{j}\cdot x_{i}+\hat{b})$$

The CAE layer is optimized through the iterative refinement of weights and errors using stochastic gradient descent. During this process, the model’s parameters are adjusted to minimize the reconstruction error, resulting in optimized feature maps. For each instance, the reconstructed output $\hat{x_{i}}$ is generated after the input undergoes both convolutional encoding and decoding. The reconstruction process involves dividing the image into patches ρ, each of size d × d, and computing the mean squared error (MSE) between the reconstructed patch $\hat{x_{i}}$ and the original input patch $x_{i}$, for *i* = 1, 2, 3, …, ρ. Equation (5) outlines the cost function, while Equation (6) provides a detailed formulation of the reconstruction error, as discussed in [41].

(6) $$JC\left(\theta \right)=\frac{1}{\rho }\sum_{i=1}^{\rho }L(x_{i},\hat{x_{i}})$$

(7) $$LC\left[x_{i},\hat{x_{i}}\right]={∥x_{i}-\hat{x_{i}}∥}^{2}={∥x_{i}-\varphi (\sigma (x_{i}))∥}^{2}$$

In the current study, an autoencoder using unsupervised learning is employed to extract fault-specific features from Mel scalograms of vibration signals, enhancing fault detection by emphasizing relevant time–frequency patterns. The Mel scalograms capture critical low-frequency information where mechanical faults are prominent, enabling the autoencoder to focus on fault-related aspects while filtering out noise. After feature extraction, a FOX-optimized ANN classifies the features, effectively distinguishing among different classes of the bearings.

### 2.3. ANN

**Figure 3:**
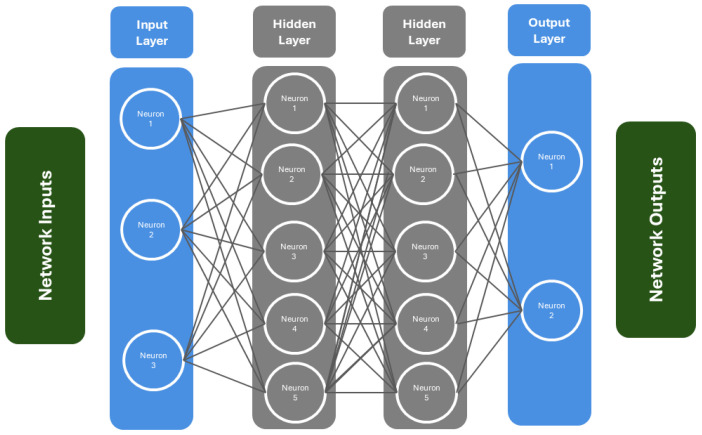
Architecture of ANN.

The ANN is composed of three primary layers: the input, hidden, and output layers, each containing multiple neurons. The input layer receives data and sends it to the hidden layer, while the output layer produces the result [42]. This flow of data from input to output is known as a feed-forward neural network. ANNs are versatile and can perform tasks such as classification, clustering, and pattern recognition. The input data are multiplied by weights and passed to the hidden-layer neurons, where an activation function computes each neuron’s output, as shown in Equation (7) and Figure 3 [30].

(8) $$a_{j}=f\left(\sum_{i=1}^{n}m_{i}\cdot m\cdot n_{ij}\right)+b_{j}\;$$

(9) $$f=\frac{1}{1+e^{x}}$$

In this context, $x_{i}$ represents the input data, $n_{ij}$ is the weight between the $i^{th}$ input neuron and the $j^{th}$ hidden neuron, b is the bias for the $j^{th}$ hidden neuron, and $a_{j}$ is the output of the $j^{th}$ neuron in the hidden layer. The neuron’s output ($a_{j}$) is then processed by an activation function, such as the sigmoid function (Equation (8)). Various activation functions, including sigmoid, tanh, ReLU, and softmax, can be used depending on the problem. For instance, the sigmoid function is suitable when the output range is between 0 and 1, whereas the tanh function is used when the output lies between −1 and 1 [42].

For optimal performance, the input data to an ANN must be standardized to reduce data variance. If the output data are in text form, it must be converted to a numerical format to be compatible with the neural network functions. Standardization and this conversion process allow the network to effectively process and analyze the data [43].

(10) $$E=\frac{1}{2}\sum_{i=1}^{n}{\left(o_{i}-{o^{\prime }}_{i}\right)}^{2}$$

(11) $$\partial_{j}=\left(o_{j}-{o^{\prime }}_{j}\right)\cdot f^{\prime }(a_{j})$$

(12) $$n_{ij}=n_{ij}+\gamma \cdot m_{i}\cdot \delta_{j}$$

In these equations, E represents the error between the expected $o_{i}$ and actual ${o^{\prime }}_{i}$ outputs, $\delta_{j}$ is the error gradient for unit $o_{j}$, $f^{\prime }(a_{j})$ is the derivative of the activation function, and $n_{ij}$ is the weight connecting unit i to j, which is updated using the learning rate γ and input $m_{i}$ [44,45].

Backpropagation is the key algorithm in an ANN, where the output is fed back into the network to adjust the weights. It comprises four main steps: First, the error between the expected and actual output is calculated using error functions like mean squared error (MSE) (Equation (9)). Second, the error gradient is derived from the outputs to update the weights in the network’s final layer (Equation (10)). Third, the error is propagated back through other layers (error backpropagation). Lastly, the weights are updated based on the derived error and learning rate (Equation (11)) [46].

### 2.4. FOX Optimizer

FOX is an optimization method inspired by the hunting behaviour of red foxes. It identifies the optimal solution using both static exploration and exploitation. During exploration, FOX employs a random walk strategy, leveraging its ultrasound detection ability to locate prey. Once the prey is detected, the agent enters the exploitation phase, evaluating the time to catch the prey based on the travel time of the ultrasound, then makes its move [47]. The FOX algorithm requires two inputs: an objective function, which calculates the fitness value, and bounds, which define the range of values for each variable in the optimization problem.

### 2.5. FOX–ANN

The proposed FOX–ANN method draws inspiration from FOX’s ability to automatically tune hyperparameters in Q-learning [48] and its superior performance in solving optimization problems. This section introduces an ANN enhanced with FOX to handle classification tasks in standard datasets, aiming to improve the network’s problem-solving capabilities. In FOX–ANN, the ANN structure remains unchanged; the key enhancement lies in replacing the backpropagation algorithm with FOX, which optimizes the weights by minimizing the MSE as per Equation (9).

**Figure 4:**
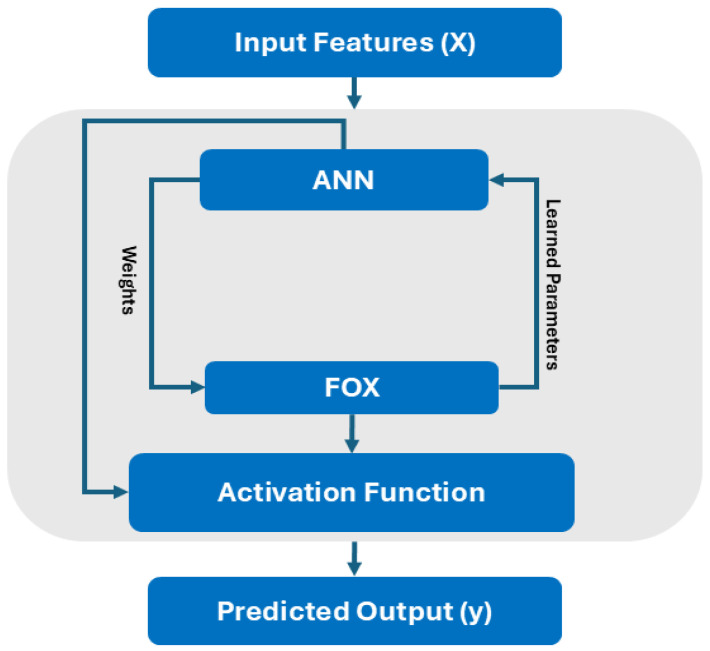
Working of FOX–ANN.

The model’s architecture is straightforward, as depicted in Figure 4. After data processing, it is directly fed into the ANN, where a feed-forward process occurs. FOX iteratively optimizes the synaptic weights to achieve minimum loss before passing them back to the ANN, which then uses these optimized weights and the activation function to compute the final output based on Equation (7).

The proposed model is structured using several layers, each serving a specific function within the overall architecture for bearing-fault classification, as shown in Table 1. It begins with an input layer, which accepts images of size (256, 256, 3). The following layers are convolutional layers (Conv2D), which apply filters to the input image to detect important features such as edges, patterns, and textures. The convolutional layers are accompanied by pooling layers (MaxPooling2D) that reduce the spatial size of the feature maps, keeping the essential information while making the processing more efficient. After extracting and refining the features through these layers, the data are passed into the decoder part of the model, which includes up sampling layers (UpSampling2D). These layers gradually increase the size of the feature maps to reconstruct the image and restore it to its original dimensions.

After the feature extraction and reconstruction are completed, the model uses a fully connected FOX–ANN for classification. The weights of this network are optimized using the FOX optimization algorithm, which adjusts weights iteratively to improve accuracy by mimicking the adaptive foraging behaviour of foxes. The flattened output from the convolutional layers is passed through several dense layers. These layers consist of multiple neurons and use activation functions like ReLU to introduce non-linearity, helping the model learn complex patterns in the data. The final dense layer, which uses the SoftMax activation function, outputs the classification results, assigning the input image to one of four bearing-fault categories.

## 3. Results and Performance Evaluation

The effectiveness of the proposed method is assessed using VS data obtained from an actual bearing testbed. Since the primary aim of this method is to detect and diagnose faults in bearings, this Section begins by comparing the fault detection capability of the proposed method.

### 3.1. Experimental Setup and Data Acquisition

**Figure 5:**
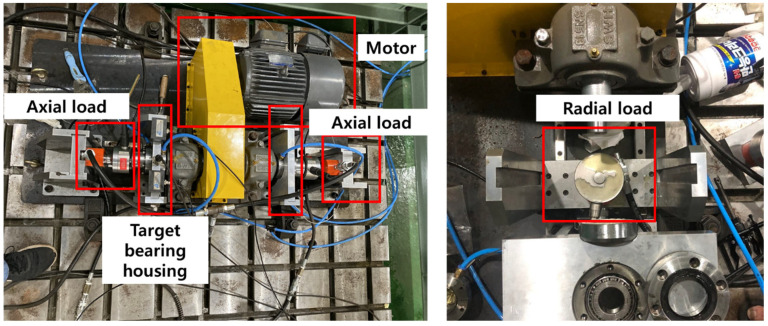
Experimental setup for bearing dataset.

**Figure 6:**
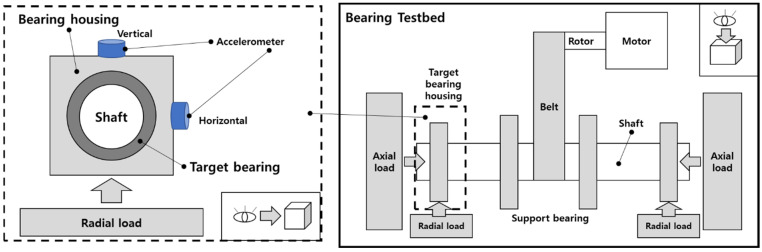
Schematic diagram of experimental setup for bearing-fault diagnosis.

Figure 5 illustrates the structure of the bearing testbed designed by the Ulsan Industrial Artificial Intelligence (UIAI) Laboratory at Ulsan University, Ulsan, South Korea, which was used in this analysis. The data collected from the bearings can be classified into four different conditions: normal, outer race fault, inner race fault, and bearings with roller fault. During the experiment, a three-phase motor drove the testbed at a fixed speed of 1800 rpm. The motion was transmitted from the rotor shaft to the main shaft by a belt installed on both sides of the testing bearings. The maximum signal was recorded from the left side of the target bearing using a vibration and AE accelerometer. The schematic diagram as shown in Figure 6 illustrates the whole experimental setup in detail.

The data acquisition system, detailed in Table 2, utilizes a FAG NJ206-3-TVP2 bearing, a cylindrical roller type. For capturing VS, an accelerometer (model PCB-622B01) was employed, while AE signals were detected using R15I-AST AE sensors. Both sensors were interfaced with an NI-9234 Data Acquisition (DAQ) device to ensure precise data collection from the Integrated Electronics Piezo-Electric (IEPE) sensors.

**Figure 7:**
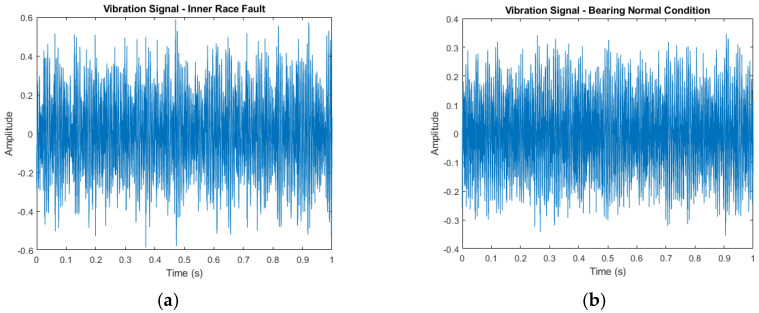

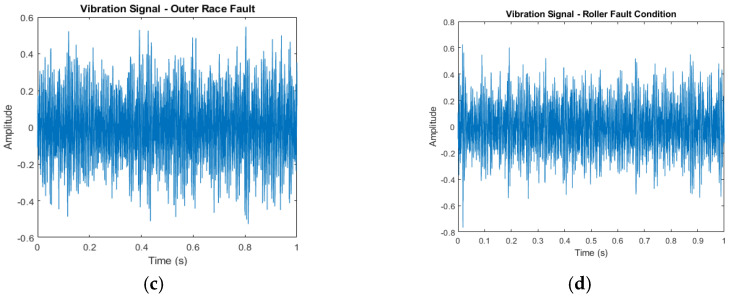
Vibration time-domain signals for different bearing-fault conditions: (**a**) IRF, (**b**) NC, (**c**) ORF, (**d**) RF.

**Figure 8:**
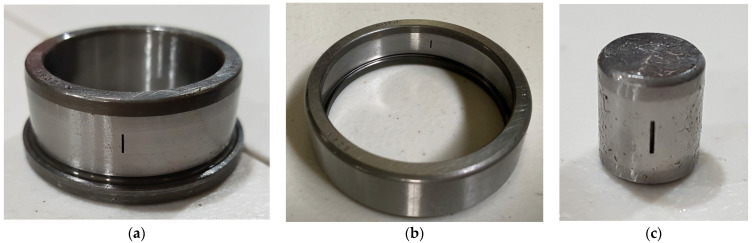
Fault components used during the experiment: (**a**) IRF, (**b**) ORF, (**c**) RF.

Vibration data were acquired at a sampling rate of 25 kHz, with five minutes of continuous data collection conducted for all bearing conditions. Subsequently, the data were divided into 1 s segments, with each 1 s segment containing 309–390 data samples for different fault types. The test procedure can be repeated for different fault types by replacing the test bearing in the same testbed setup. Figure 7 represents the time domain signals of the bearing in the four conditions where as Figure 8 shows the bearings used for collection of the data set clearly showing the defects. 

### 3.2. Performance Metrics for Comparisons

In this study, the proposed model demonstrates robust performance using Mel-transformed scalograms generated from VS of bearings. The process begins with truncating signals using windowing techniques, followed by applying a Mel filter bank to convert the signals into a Mel spectrum. These Mel scalograms are then processed through the convolutional and pooling layers of an autoencoder, enabling effective feature extraction from the transformed spectrum. The extracted features are classified using a FOX optimizer with an ANN which optimizes synaptic weights more efficiently than traditional backpropagation. The FOX optimizer enhances model accuracy, reduces loss, and improves both generalization and interpretability. To validate the proposed approach, a laboratory dataset collected from a bearing testbed with multiple fault conditions was used. The experimental results illustrate the model’s robustness and generalizability, showing its effectiveness in fault diagnosis tasks across various bearing faults. These findings confirm that the integration of Mel-transformed scalograms and the FOX optimizer contributes significantly to the model’s high-performance capabilities.

The evaluation of the proposed method is conducted using key performance metrics, including accuracy, precision, recall, and F1-score, as outlined in Equations (12)–(15). These metrics are essential for measuring the model’s ability to accurately classify different fault conditions in the milling machine. By leveraging this comprehensive evaluation approach, the proposed model is thoroughly assessed for its capability in real-world fault detection and diagnosis, ensuring its suitability for industrial applications. This thorough evaluation confirms the model’s reliability and effectiveness in practical fault classification tasks.

(13) $$Accuracy=\frac{\left(TN+TP\right)}{\left(TP+TN+FP+FN\right)}\times 100\%$$

(14) $$Precision=\frac{TP}{TP+FP}\times 100\%$$

(15) $$Recall=\frac{TP}{TP+FN}\times 100\%$$

(16) $$F1-Score=\frac{2TP}{2TP+FP+FN}=\frac{2\times (Precision\times Recall)}{Precision+Recall}$$

Here, true positives (TPs) represent instances where the model correctly classifies faulty conditions, while true negatives (TNs) refer to cases accurately identified as non-faulty. false positives (FPs) occur when the model mistakenly identifies non-faulty instances as faulty, and false negatives (FNs) represent faulty instances incorrectly classified as non-faulty. These metrics offer a comprehensive assessment of the model’s classification performance, ensuring its robustness and accuracy in detecting and diagnosing bearing faults. The integration of FOX–ANN for classification enhances the model’s ability to effectively differentiate between various fault types, resulting in improved diagnostic precision and reliability.

### 3.3. Comparative Analysis of Fault Diagnosis Methods

To evaluate the effectiveness of the proposed fault detection method, we compare it with two other recent methods: Zhang et al. [49] and Guanghua Fu et al. [50]. The performance of each method is assessed based on the matrices provided in Equations (8)–(11). The results obtained from the proposed and reference methods are presented in Table 3, while the per-class true-positive rate is presented in Table 4.

**Figure 9:**
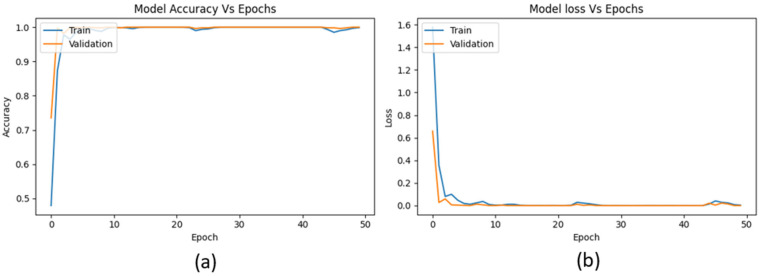
Proposed model: (**a**) accuracy vs. number of epochs and (**b**) losses vs. number of epochs.

The proposed bearing-fault diagnosis model incorporates several advanced techniques to enhance fault detection and classification. It begins by capturing VS from the bearing system, which are processed and converted into Mel-transformed scalograms. This transformation leverages a Mel filter bank to convert the signals into a Mel spectrum, providing a time–frequency representation that captures essential fault-related features. These scalograms are then fed into the convolutional and pooling layers of an autoencoder, which extracts meaningful features from the Mel spectrum. The extracted features are then classified using an ANN, where a FOX optimizer is employed instead of traditional backpropagation. This optimizer fine-tunes synaptic weights, leading to high accuracy, minimal loss, improved generalization, and enhanced interpretability of the model. The approach is validated using a bearing dataset collected from a laboratory testbed with multiple bearing fault conditions, including IRF, ORF, RF, and NCs. The accuracy vs. epochs graph for the proposed model as depicted in Figure 9 shows that both training and validation accuracy quickly converge near 1.0 within the first few epochs, indicating rapid learning and consistent performance across the dataset. Meanwhile, the loss vs. epochs graph demonstrates a sharp drop in both training and validation loss early on, stabilizing near zero, signifying effective minimization of errors. These graphs reflect the model’s high performance and stability, with no signs of overfitting as both training and validation curves align closely.

**Figure 10:**
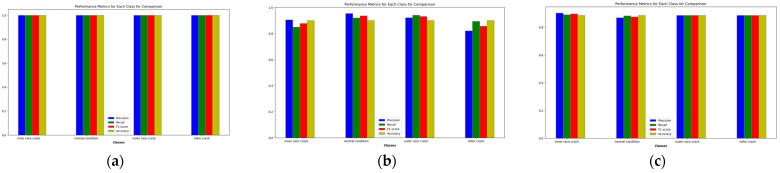
Performance metrics comparison of the (**a**) proposed model, (**b**) Zhang et al., and (**c**) Fu et al.

**Figure 11:**
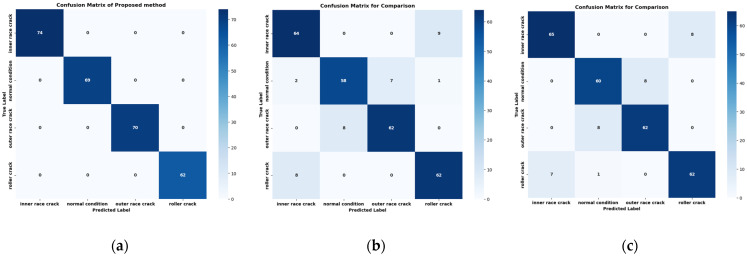
Confusion matrices comparison of the (**a**) proposed model with (**b**) Zhang et al. and (**c**) Fu et al.

**Figure 12:**
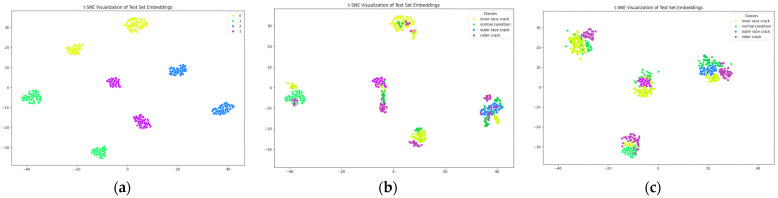
t-SNE comparison of the (**a**) proposed model with (**b**) Zhang et al. and (**c**) Fu et al.

**Figure 13:**
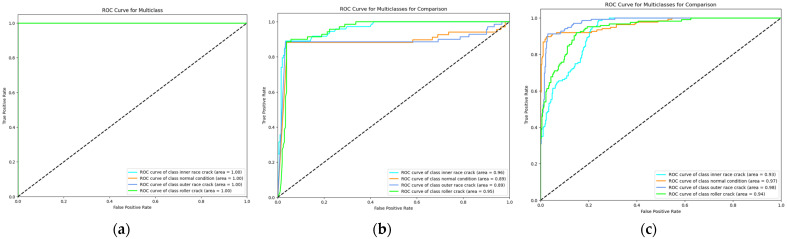
ROC curves comparison of the (**a**) proposed model with (**b**) Zhang et al. and (**c**) Fu et al.

The proposed method demonstrates outstanding performance across several key metrics. As shown in Table 4, the classification report presents perfect precision, recall, and F1-scores of 1.00 for each fault class, including IRF, NC, ORF, and RF, as summarized in Table 5. Figure 10 shows the performance scores of the proposed and comparison models on a bar plot. This is further validated by the confusion matrix in Figure 11, which shows no misclassifications, with all true labels accurately predicted. Specifically, the confusion matrix reflects 74/74 correct predictions for IRF, 69/69 for NC, 70/70 for ORF, and 62/62 for RF, confirming the model’s precision and recall of 1.00 across all classes. Figure 12 represents the t-SNE plots. The ROC curve in Figure 13 supports these results, displaying an AUC of 1.00 for each fault class, highlighting the model’s excellent capability in distinguishing between different fault conditions with no false positives or negatives. Additionally, the performance metrics bar chart confirms consistently high values for precision, recall, F1-score, and accuracy across all classes, underscoring the model’s robustness and reliability. These results showcase the effectiveness of the proposed method in accurately detecting and classifying various bearing faults. By leveraging Mel-transformed scalograms and the FOX optimizer integrated with an ANN, the model not only enhances classification accuracy but also improves generalization and interpretability. The classification performance across all metrics underscores the model’s suitability for real-world applications, particularly in predictive maintenance and fault diagnosis for industrial bearing systems. The t-SNE plot further illustrates the model’s ability to clearly differentiate between fault classes, with distinct and well-separated clusters for IRF, NC, ORF, and RF as shown in Figure 12. This strong class separability reinforces the model’s high classification performance and robustness.

In comparison, the model proposed by Zhang et al. [49] which employs a combination of STFT and a CNN for bearing fault detection, converts raw 1D VS into 2D time–frequency scalograms for classification. When applied to our dataset, Zhang et al.’s model demonstrated a strong overall performance, achieving an accuracy of 91.2% across the four fault categories, as shown in Table 4 and Figure 10. However, in confusion matrices, some misclassifications are present. The model correctly classified 64 out of 73 IRF samples, but 9 were misclassified as RF, leading to a precision of 0.89 and recall of 0.94. The NC class saw 58 out of 68 samples correctly classified, though 7 were misclassified as ORF and 1 as RF, resulting in a precision of 0.90 and recall of 0.85. The ORF class correctly identified 62 out of 70 samples, but 8 were misclassified as NC, yielding a precision of 0.89 and recall of 0.89. Lastly, the RF class was correctly classified in 62 out of 70 instances, but 8 samples were misclassified as IRF. While the model shows strong diagnostic capability, the confusion between IRF and RF categories suggests the need for improved feature extraction techniques to better distinguish between these closely related fault types as illustrated in Figure 11. The t-SNE plot for Zhang et al.’s model indicates a similar trend, with noticeable overlap between these two classes, implying potential refinements are necessary for better separability as shown in Figure 12. The ROC curves further reflect these challenges, showing AUC values of 0.96 for IRF, 0.97 for NC, 0.98 for ORF, and 0.94 for RF, as shown in Figure 13, indicating solid performance overall but room for improvement in fault differentiation between closely related categories.

Similarly, the model by Guanghua Fu et al. [50] integrates CNN and Bi-LSTM with a residual module to address bearing-fault diagnosis challenges. This model employs a dual-path feature extraction mechanism where Bi-LSTM extracts temporal features from VS, and CNN processes spatial features from time–frequency images. These features are then fused, and a residual module enhances feature extraction, particularly improving performance in noisy environments. When compared to the baseline model and applied to our dataset, Fu et al.’s model achieved an overall accuracy of 91.02%, with precision, recall, and F1-scores of 91.50%, as summarized in Table 4 and Figure 10. However, there were notable misclassifications. The confusion matrix, as shown in Figure 11, reveals that the model correctly classified 65 IRF samples, but misclassified 8 as RF. For the NC class, it correctly classified 60 out of 68 samples, while 8 were misclassified as ORF. Similarly, the ORF class saw 62 correct classifications, but 8 were misclassified as NC. Lastly, the RF class showed 62 correct classifications, but 7 samples were confused with IRF. Comparatively, the proposed model significantly outperforms Fu et al.’s model in terms of accuracy, particularly in differentiating closely related faults like inner race and RFs. The t-SNE visualization, as shown in Figure 12 for Fu et al.’s model, shows clearer separations between fault categories than Zhang et al.’s model but still struggles with overlap between inner race and RFs, a challenge that the proposed model successfully overcomes. Finally, the ROC curves, as illustrated in Figure 13, further highlight the superior diagnostic ability of the proposed model. The AUC values for all fault classes in the proposed model exceeded 0.93, compared to Fu et al.’s model, which showed lower AUCs, particularly for more challenging fault types such as inner race and RFs.

Finally, the proposed method demonstrates better performance across all classes as shown in Table 5, achieving a true positive rate (TPR) of 100% for IRC, NC, ORC, and RF, indicating classification for all fault types. In comparison, Zhang et al.’s model shows slightly lower performance, with TPRs of 87.67% for IRC, 85.29% for NC, 88.57% for ORC, and 100% for RF. Fu et al.’s method performs better than Zhang et al.’s in IRC and NC classification with TPRs of 89.04% and 88.24% but matches Zhang et al.’s TPR for ORC (88.57%) and is slightly lower for RF at 88.57%.

Overall, the proposed method outperforms both Zhang et al. and Fu et al. in terms of fault classification, particularly excelling in NC and RF where the other models show limitations. Overall, the proposed model’s improved performance across all metrics underscores its effectiveness in feature extraction, handling noise, and accurately classifying faults, making it a more reliable and robust solution for bearing-fault diagnosis compared to the models by Zhang et al. and Fu et al. This research makes the way for improved predictive maintenance and operational efficiency in industrial settings.

## 4. Conclusions

The proposed Mel-transformed scalogram-based model for bearing-fault diagnosis demonstrates exceptional performance, achieving perfect classification metrics such as precision, recall, and F1-scores across all fault categories. The integration of the FOX optimizer with an ANN replaces traditional backpropagation, optimizing synaptic weights effectively to enhance both accuracy and generalization. Comparative analysis with models by Zhang et al. and Fu et al. reveals that the proposed approach significantly outperforms them, particularly in distinguishing closely related fault types, such as inner race and roller faults. The model’s robustness in noisy environments and its ability to clearly separate fault classes in t-SNE plots further emphasize its practical utility. Moreover, the use of Mel-transformed scalograms provides richer feature extraction from VS, contributing to improved diagnostic accuracy. Overall, the method proves to be a reliable and scalable solution for real-world predictive maintenance in industrial applications, offering both robustness and high generalizability across diverse fault conditions.

Future works could focus on expanding the proposed approach to more complex industrial datasets with additional fault types and varying operational conditions. Furthermore, analyzing how specific defects influence signal features within Mel-scaled representations is essential to deepen the understanding of fault characteristics—such as the harmonic patterns of inner race faults and the high-amplitude bursts of roller faults. A hybrid approach using both Mel filter banks and wavelet transforms could also be explored, as Mel filters are effective for capturing low-frequency components, while wavelet transforms can identify high-frequency transients and non-stationary characteristics, providing a more comprehensive signal analysis. One limitation of this study is the reliance on a simplified envelope analysis model for benchmarking, which may not capture the full range of potential in envelope-based fault detection. Incorporating real-time data streaming and integrating the model with edge computing systems for on-site predictive maintenance could enhance its practical application in industrial settings.

## Figures and Tables

**Table 1:** Proposed-model summary.

	Type of Layer	No. of Filters/Neurons	Kernel Size	Output Shape	Activation Function
0	Input Layer	-	-	(None, 256, 256, 3)	-
1	Conv2D	32	(3, 3)	(None, 256, 256, 32)	ReLU
2	MaxPooling2D	-	-	(None, 128, 128, 32)	-
3	Conv2D	64	(3, 3)	(None, 128, 128, 64)	ReLU
4	MaxPooling2D	-	-	(None, 64, 64, 64)	-
5	Conv2D	128	(3, 3)	(None, 64, 64, 128)	ReLU
6	MaxPooling2D	-	-	(None, 32, 32, 128)	-
7	InputLayer	-	-	(None, 32, 32, 128)	-
8	Conv2D	128	(3, 3)	(None, 32, 32, 128)	ReLU
9	UpSampling2D	-	-	(None, 64, 64, 128)	-
10	Conv2D	64	(3, 3)	(None, 64, 64, 64)	ReLU
11	UpSampling2D	-	-	(None, 128, 128, 64)	-
12	Conv2D	32	(3, 3)	(None, 128, 128, 32)	ReLU
13	UpSampling2D	-	-	(None, 256, 256, 32)	-
14	Conv2D	3	(3, 3)	(None, 256, 256, 3)	softmax
15	Flatten	-	-	(None, 131072)	-
16	Dense	512	-	(None, 512)	ReLU
17	Dense	256	-	(None, 256)	ReLU
18	Dense	128	-	(None, 128)	ReLU
19	Dense	4	-	(None, 4)	softmax

**Table 2:** Specifications of the data-acquisition system.

Device	Specification	Value
Vibration sensor (PCB-622B01)	Measurement range	±490 m/s^2^
	Frequency	0.2–15,000 Hz
	Sensitivity	100 mV/g
AE sensor (R151-AST)	Operating range	50–400 kHz
	Resonant frequency	150 kHz
	Peak sensitivity	−22 dB
DAQ (NI 9234)	Dynamic range	102 dB
	Resolution	24-bit
	Operating temperature	−40 °C–70 °C

**Table 3:** Data acquisition summary.

Testing Condition	Samples Count	Sampling Rate (KHz)	Time (min)
IRF	370	25	5
NC	390	25	5
ORF	347	25	5
RF	309	25	5

**Table 4:** Performance comparison of comparative methods with the proposed method.

Performance Metrics	Proposed	Zhang et al. [49]	Fu et al. [50]
Accuracy (%)	99.93	92.03	91.02
Precision (%)	100	92.25	91.50
F1 Score (%)	100	92.50	91.50
Recall (%)	100	92.00	91.25

**Table 5:** TPR comparison of comparative methods with the proposed method.

Models	IRF	NC	ORF	RF
Proposed	100	100	100	100
Zhang et al. [49]	87.67	85.29	88.57	100
Fu et al. [50]	89.04	88.24	88.57	88.57

## Data Availability

Data will be provided upon request.

## Nomenclature

STFT	Short-Time Fourier Transform
CWT	Continuous Wavelet Transform
IRF	Inner Race Fault
ORF	Outer Race Fault
RF	Roller Fault
NC	Normal Condition
CNN	Convolutional Neural network
Bi-LSTM	Bidirectional Long Short Time Memory
ReLU	Rectified Linear Unit
TPR	True Positive Rate
ANN	Artificial Neural Network
